# 

**Published:** 1895-08-10

**Authors:** 


### CONTENTS.

- **Drugs Many: Remedies Few** - 317

- **The Cycle of Life** - 318

- **On Modern Progress in Ophthalmic Medicine and Surgery.** By Robert Brudenell Carter, F.R.F.S. - 319

- **Moral Mania** - 320

- **Annotations.**—The Morals of a Man of Science—The Doctor and the Lawyer: An Ancient Conflict—The Throat Hospital, Golden Square - 321

- **Notes and News** - 322

- **British Medical Association.**—Proceedings of Sections - 323

- **Medical Progress and Hospital Clinics**—

  - Unsuspected Thoracic Aneurism - 327

- **The Institutional Workshop**—

  - Hospital Administration.—The Queen's Jubilee Hospital - 329

  - British Medical Association - 330

  - Hospital Festivals and Meetings - 331

- **Editor's Letter Box** - 332

- **Scraps and Gleanings** - 332

- **The Book World of Medicine and Science** - 333

- **The Student of Medicine** - 333

### EXTRA SUPPLEMENT.—THE NURSING MIRROR.

- **News from the Nursing World**—Our Princess—Hats On and Off—The Nurses' Thanks—Killed by Ignorance—Nursing of Outdoor Patients—Who was the Nurse?—Macclesfield—Training at Bristol—Successful Organization—Omissions Quickly Rectified—Nurses at Sunderland—Progress at Wigan—Efficient, But Not Sufficient—Nurses' Homes—More Subscriptions Required - cxxix

- **Elementary Physiology for Nurses.** By C. F. Marshall, M.D., B.Sc., F.R.C.S. I.—Introduction - cxxxi

- **"The Hospital" Convalescent Fund** - cxxxi

- **Training for Lecturers** - cxxxi

- **Our Princess's Garden Party** - cxxxii

- **Where to Go** - cxxxii

- **Holidays and Health** - cxxxiii

- **Everybody's Opinion** - cxxxiv

- **Trained Nurses at Bradford** - cxxxiv

- **A Hospital Ship** - cxxxiv

- **Appointments—Notes and Queries** - cxxxiv

